# Process evaluation of a participatory ergonomics programme to prevent low back pain and neck pain among workers

**DOI:** 10.1186/1748-5908-5-65

**Published:** 2010-08-24

**Authors:** Maurice T Driessen, Karin I Proper, Johannes R Anema, Paulien M Bongers, Allard J van der Beek

**Affiliations:** 1 Body@Work TNO VUmc, Research Center Physical Activity, Work and Health, VU University Medical Center, van der Boechorststraat 7, 1081 BT Amsterdam, The Netherlands; 2 Department of Public and Occupational Health, EMGO Institute for Health and Care Research, VU University Medical Center, van der Boechorststraat 7, 1081 BT Amsterdam, The Netherlands; 3 TNO Quality of Life, Polarisavenue 151, 2132 JJ, Hoofddorp, The Netherlands

## Abstract

**Background:**

Both low back pain (LBP) and neck pain (NP) are major occupational health problems. In the workplace, participatory ergonomics (PE) is frequently used on musculoskeletal disorders. However, evidence on the effectiveness of PE to prevent LBP and NP obtained from randomised controlled trials (RCTs) is scarce. This study evaluates the process of the Stay@Work participatory ergonomics programme, including the perceived implementation of the prioritised ergonomic measures.

**Methods:**

This cluster-RCT was conducted at the departments of four Dutch companies (a railway transportation company, an airline company, a steel company, and a university including its university medical hospital). Directly after the randomisation outcome, intervention departments formed a working group that followed the steps of PE during a six-hour working group meeting. Guided by an ergonomist, working groups identified and prioritised risk factors for LBP and NP, and composed and prioritised ergonomic measures. Within three months after the meeting, working groups had to implement the prioritised ergonomic measures at their department. Data on various process components (recruitment, reach, fidelity, satisfaction, and implementation components, *i.e.*, dose delivered and dose received) were collected and analysed on two levels: department (*i.e.*, working group members from intervention departments) and participant (*i.e.*, workers from intervention departments).

**Results:**

A total of 19 intervention departments (n = 10 with mental workloads, n = 1 with a light physical workload, n = 4 departments with physical and mental workloads, and n = 4 with heavy physical workloads) were recruited for participation, and the reach among working group members who participated was high (87%). Fidelity and satisfaction towards the PE programme rated by the working group members was good (7.3 or higher). The same was found for the Stay@Work ergocoach training (7.5 or higher). In total, 66 ergonomic measures were prioritised by the working groups. Altogether, 34% of all prioritised ergonomic measures were perceived as implemented (dose delivered), while the workers at the intervention departments perceived 26% as implemented (dose received).

**Conclusions:**

PE can be a successful method to develop and to prioritise ergonomic measures to prevent LBP and NP. Despite the positive rating of the PE programme the implementation of the prioritised ergonomic measures was lower than expected.

**Trial registration:**

Current Controlled Trials ISRCTN27472278

## Background

The prevalence of low back pain (LBP) and neck pain (NP) among workers is high [1,2]. To prevent or reduce these symptoms, ergonomic interventions are commonly applied [3]. However, ergonomic interventions appeared to be most often not effective in the prevention of LBP and NP [2,4-6]. An important reason for finding no effects on LBP and NP might be due to the inadequate implementation of ergonomic measures (*i.e.*, compliance, satisfactions and experiences) and the lack of using adequate implementation strategies [7].

Participatory ergonomics (PE) is a noted implementation strategy to develop ergonomic measures from the bottom up [8-10]. According to the stepwise PE method, ergonomic measures are developed by working groups (consisting of workers, management, and other important stakeholders) [8,10-12]. By using this bottom up approach, the acceptance to use the ergonomic measures may become more widespread among end-users (*i.e.*, workers). To inform, educate, and instruct workers on the PE process, other supportive implementation strategies, such as distribution of brochures and flyers, providing training, and capitalising on opinion leaders are used [13,14]. The actual implementation of ergonomic measures is considered as a (possible) consequence of the PE process and can be enhanced by the use of additional implementation strategies (*e.g.*, use of opinion leaders).

The effects of PE on the reduction of musculoskeletal disorders (MSD) have shown to be promising [15-21]. However, it should be noted that most studies on the effectiveness of PE were of low quality and were conducted in a working population with heavy workloads. Studies directly assessing the prevention of MSD are rare, especially those using a randomised study design. The only randomised controlled trial (RCT) in the area of PE and the prevention of MSD has been conducted by Haukka *et al. *(2008). They showed that PE was not effective to prevent MSD among kitchen workers [22]. More high-quality studies (RCTs) evaluating the effectiveness of PE are needed. Therefore, "The Stay@Work study" currently investigates the effectiveness of a PE programme on the prevention of LBP and NP among a heterogeneous population of workers [23].

In the past years, the conduct of process evaluations alongside RCTs has been recommended, because they can facilitate the interpretation of the findings [24]. For example, a process evaluation can shed light on whether the intervention was delivered as intended (*i.e.*, compliance, adherence, satisfaction, and experiences) as well as the success and failures of the intervention programme [25-28]. Moreover, the information obtained from a process evaluation can be used to further improve the intervention [26,29], and to enable the transition of research evidence into occupational health practice [30]. Therefore, this study evaluated the process of the Stay@Work PE programme, including the perceived implementation of the prioritised ergonomic measures.

## Methods

This process evaluation was performed alongside a RCT on the effectiveness of a PE programme on the prevention of LBP and NP among workers, called Stay@Work. The Medical Ethics Committee of the VU University Medical Center approved the study protocol. Detailed information on the methods, randomisation procedure, and intervention can be found elsewhere [23]. The departments of four large Dutch companies (a railway transportation company, an airline company, a university including its university medical hospital, and a steel company) were invited to participate in the study. The higher management of all companies agreed with the financial and organisational consequences of the intervention. Based on their main workload, participating departments were classified into: mental, physical, mix mental/physical, or heavy physical departments [31]. Within each company, one randomisation pair of two departments with comparable workloads was randomly allocated to either the intervention group (Stay@Work PE programme) or the control group (no Stay@Work PE programme).

All workers at the departments of both groups received the baseline questionnaire and watched three short (45 seconds) educative movies about the prevention of LBP and NP.

### The Stay@Work PE programme

In short, the intervention comprised a six-hour working group meeting, in which the steps of the Stay@Work PE programme were followed. Each intervention department had to form a 'working group', in which both workers and management participated as members [8,11]. Each working group consisted of at least one manager with decision authority, a maximum of eight workers who were a solid representation of the largest and most important task groups at the department. If available, an occupational health and safety coordinator was incorporated in the working group as well. Working group members had to have worked at least two years in their current job, worked for more than 20 hours per week at the department, had responsibilities within his/her own task group, was a role model for his/her co-workers, and was motivated to participate as a member in the working group [23]. During the first meeting, the working group discussed a document containing information on risk factors on LBP and NP present at the department, which were obtained from the ergonomist workplace visit (which was mandatory for each intervention department), pictures made by the working group members, and baseline questionnaire information (step one). Then, the working group could add other risk factors of LBP and NP, and judged all mentioned risk factors as to their frequency and severity. Based on the perceptions of the working group, the most frequent and severe risk factors were prioritised, resulting in a top three of risk factors (step two). Subsequently, the working group held a brainstorming session about different types of ergonomic measures targeting the prioritised risk factors, evaluated the ergonomic measures according to a criteria list considering: relative advantage, costs, compatibility, complexity, visibility, and feasibility within a time frame of three months [32]. On a consensus basis, the working group prioritised the three most appropriate ergonomic measures (step three). Finally, the prioritised risk factors and the prioritised ergonomic measures were written down in an implementation plan (step four). The implementation plan described for each ergonomic measure which working group members were responsible for its implementation. Based on their interests in the projects, the prioritised ergonomic measures were divided among the members of the working group. Working group members who had a responsibility towards implementation of a prioritised ergonomic measure were called the 'implementers.' At the end of the meeting, the working group was requested to implement the ergonomic measures (step five) and was asked whether an appointment for a second, optional, meeting was necessary to evaluate or adjust the implementation process (step six). During the implementation process, all working groups were allowed to ask help from other professionals (*i.e.*, technicians, engineers, or suppliers) or services (*i.e.*, equipment or health services). To improve the implementation process, two or three working group members from each working group were asked to voluntarily follow a training programme to become a Stay@Work ergocoach. In this additional four-hour implementation facilitation training, workers were educated in different implementation strategies to inform, motivate, and instruct co-workers about the prioritised ergonomic measures. Moreover, the ergocoaches were equipped with a Stay@Work toolkit consisting of flyers, posters, and presentation formats about the prioritised ergonomic measures. According to the Attitude - Social influence - self-Efficacy (ASE) behavioural change model that was applied during the PE programme, dissemination of information about ergonomic measures may increase worker's self-awareness of their own behaviour and increase knowledge about possible ergonomic solutions. Thus informing workers can be regarded as a first step in order to induce a behavioural change [13,33].

### The process evaluation

An adapted version of the Linnan and Steckler framework, which has been recommended to be a useful guide for the conduct of a process evaluation, was used [34,35]. Table 1 presents the components that were addressed; recruitment, reach, fidelity, satisfaction, and implementation components (*i.e.*, dose delivered and dose received).

**Table 1:** Process evaluation components and their definitions

Component	Definition
Recruitment	- Number of intervention departments that agreed to participate
	- Number of working groups formed
	- Number of working group members recruited for additional ergocoach training
	- Number of workers who responded to the baseline questionnaire

Reach	- Number of worksite visits by ergonomist
	- Number of working group members who attended working group meeting
	- Number of working group members who attended the Stay@Work ergocoach training

Fidelity	- The extent to which the steps of the PE programme were delivered as intended

Satisfaction	- Satisfaction of working group members towards the prioritised risk factors and ergonomic measures, the ergonomist's competences, and duration of the working group meeting
	- Satisfaction of working group members who followed the Stay@Work ergocoach training towards the course leader's competences, and the duration of the training
	- Satisfaction of workers at the department towards the perceived implemented ergonomic measures and towards the intervention method (PE) that was used to develop the ergonomic measures

Dose delivered	- Perceived implementation of the ergonomic measures according to the implementers

Dose received	- Perceived implementation of the prioritised ergonomic measures according to the workers at the departments
	- Workplace implementation of the prioritised ergonomic measures according to the workers at the departments

### Data collection

The process evaluation was conducted for the intervention departments only. The PE programme is a complex intervention, containing components that may affect different levels. Therefore, if appropriate, data on the components were collected on two levels (see Table 2): department level (*i.e.*, working group members from intervention departments) and participant level (*i.e.*, workers from intervention departments).

**Table 2:** Process evaluation data collection: main levels and methods

Component	Department level	Participant level	Data collection tool
Recruitment	X	X	Checklist and baseline questionnaire

Reach	X		Checklist

Fidelity	X		1 to 10 scale (very bad to very good)

Satisfaction	X	X	1 to 10 scale (very unsatisfied to very satisfied)

Dose delivered	X		Questionnaire assessing for each prioritised ergonomic measure the perceived implementation (yes/partly/no)

Dose received		X	Questionnaire assessing for each prioritised ergonomic measure the:
			1) Perceived implementation (yes/no/don't know)
			2) Workplace implementation (yes/no)

### Recruitment

#### Department level recruitment

The department level was defined as the number of intervention departments that agreed to participate in the study and the number of working groups formed. Managers who formed the working group had to send a list with names of the working group members to the principal researcher. At the end of each working group meeting, two or three members were recruited for the additional Stay@Work ergocoach training.

#### Participant level recruitment

The level of the participant was defined as the number of workers who filled out the baseline questionnaire.

### Department level reach

At the level of the department, 'reach' was defined in two ways. First as the number of worksite visits conducted by the ergonomists. During a worksite visit, the ergonomist observed activities or situations that were considered relevant for LBP and NP. Information on the workplace visits was sent to the principal researcher. Second, reach was defined as the number of workers that attended the working group meeting and the number of working group members that attended the Stay@Work ergocoach training. Before the start of each session, all working group members had to sign a list to confirm their attendance. Reasons for not attending were registered.

### Department level fidelity and satisfaction

Directly after finishing the working group meeting, all working group members were asked to report on the components fidelity and satisfaction: at the level of the department, 'fidelity' was defined as the extent to which the steps of the PE programme were delivered as intended, and was rated on an 1-10 point scale (very bad to very good); at the level of the department, 'satisfaction' was rated on an 1-10 point scale (very unsatisfied to very satisfied) and encompassed satisfaction towards the outcomes (risk factors and ergonomic measures prioritised), the ergonomist's competences, and the duration of the meeting was assessed. By using the same components (fidelity and satisfaction) and measures (1-10 scale), the Stay@Work ergocoach training was evaluated.

### Participant level satisfaction

At the level of the participant, satisfaction could only be measured among workers who perceived at least one ergonomic measure as implemented. By using an 1-10 point scale (very unsatisfied to very satisfied), satisfaction with the perceived implemented ergonomic measure(s) was assessed; likewise, satisfaction with the intervention method (PE) used to develop ergonomic measures was measured. These workers were also asked on how they took notice of the supportive implementation measures (*i.e.*, e-mail/poster/flyer).

### Implementation

#### Department level dose delivered

Four months after finishing the working group meeting, the implementers -- working group member(s) responsible for the implementation of one or more prioritised ergonomic measure(s) -- received a short questionnaire. Implementers were asked whether the prioritised ergonomic measures for which he/she was responsible for were realised (implemented) at the department as described in the original implementation plan. The perceived implementation was assessed separately for each ergonomic measure. For each ergonomic measure, the implementers could choose from three answer categories:

1. yes, implemented: the prioritised ergonomic measure was realised as described in the implementation plan.

2. yes, partly implemented.

3. no, not implemented: the prioritised ergonomic measure was not realised as described in the implementation plan.

This method enabled the investigators to calculate for each ergonomic measure of interest a percentage of the perceived implementation. The implementation percentage was derived by summing the frequencies of each of the three answer categories (yes, implemented/yes, partly implemented/no, not implemented). By summing all implementation percentages and dividing by the total number of prioritised ergonomic measures, an overall implementation percentage for all departments could be calculated.

#### Participant level dose received

All information on the participant level was obtained from workers who responded to the six-month follow-up questionnaire, and addressed information on:

1. The perceived implementation of the ergonomic measures was measured by means of a separate question that asked workers whether the prioritised ergonomic measure was implemented by the working group at their department. For each ergonomic measure, three answers were possible: yes/no/don't know. By using a procedure similar to the one for dose delivered, an overall perceived implementation percentage was calculated.

2. The workplace implementation was assessed among those workers who perceived an ergonomic measure as implemented. By means of another question they were asked whether the ergonomic measure was applicable to their workplace (yes/no). The percentage of implemented measures at their workplace was derived by dividing the number of 'yes actually implemented' by the number of 'yes perceived as implemented'.

## Results

### Recruitment and reach

#### Department level

In total, 37 departments were included in the randomisation procedure with 19 departments randomised to the intervention group. Among the intervention departments, 10 departments were characterised by mental workloads, one department had a light physical workload, four departments had mixed workloads (physical and mental), and four departments had heavy physical workloads.

One department with a mixed workload (n = 103 workers) dropped out of the study due to a sudden reorganisation, and no working group was formed at that department. Further, as the department managers of four departments with a 'mental workload' were not able to select a sufficient number of workers to participate in the working group, it was decided to form two working groups instead of four. Thus, out of 18 departments, 16 working groups were formed. In total, 113 working group members were invited to participate. All working groups held a working group meeting, which was attended by 98 working group members (87%). Of the 15 non-attending members six were on sick leave, seven were too busy, one had a regular day off, and one was no longer working at the department.

Eight Stay@Work ergocoach training sessions were held and were attended by 40 working group members. The number of members per working group that followed the training varied from one to six.

#### Participant level

The baseline questionnaire was sent to 5,695 workers, of whom 3,232 (57%) responded. A total of 185 workers did not meet the inclusion criteria for data analyses, which were: aged between 18 years and 65 years; no cumulative sick leave period longer than four weeks due to LBP or NP in the past three months before the start of the intervention; and not pregnant [23]. Hence, at baseline 3,047 (53%) workers were included. Among them, 1,472 workers were working at intervention departments. Compliance to watching the movies on LBP and NP prevention in the intervention group was 67%.

### Fidelity and satisfaction

#### Department level

Six trained ergonomists conducted the worksite visits (n = 18) and guided the working group meetings. The number of working groups that each ergonomist guided varied from one to five.

All 16 working groups completed the first working group meeting according to the study protocol and developed an implementation plan. Three working groups, all characterised by heavy physical workloads, planned the second (optional) working group meeting. Working group members (n = 98) rated the quality of the PE steps performed between 7.32 (SD 1.02) and 7.59 (SD 0.99), and were satisfied with the risk factors and ergonomic measures prioritised (7.30, SD 1.15), the ergonomist's competences (7.70, SD 0.92) and the six-hour duration of the meeting (7.06, SD 1.30).

In total, 40 working group members (25 men and 15 women) followed the Stay@Work ergocoach training and were positive about the quality of the training (7.67, SD 0.48), were satisfied with the course leader's competences (8.03, SD 0.70), and with the four-hour duration of the training (7.53 (SD 1.15)).

#### Participant level

Workers at the departments who perceived at least one of the ergonomic measures as implemented were informed about the ergonomic measure(s) by poster/flyer/e-mail (55%), by a presentation provided by a working group member (41%), or by their supervisor (24%). Workers rated their satisfaction towards the ergonomic measures as prioritised by the working group (5.72, SD 2.39) and the method (PE) used to develop and prioritise the ergonomic measures (5.59, SD 2.29). In case the ergonomic measures were implemented at their workplace, satisfaction towards the ergonomic measures was 6.02 (SD 2.31). For the method used to develop and prioritise the ergonomic measures their satisfaction was 5.82 (SD 2.23).

### Implementation

#### Department level: dose delivered

In total, the working groups prioritised 66 ergonomic measures. The number of ergonomic measures per working group varied from three to six. The 66 prioritised ergonomic measures were classified by two researchers independently from each other into three categories: individual, physical, and organisational ergonomic measures [36]. The classification resulted in: 32 individual, 27 physical, and 7 organisational ergonomic measures (see Table 3).

**Table 3:** Types and targets of the prioritised ergonomic measures (n = 66)

Type of ergonomic measure	Target of ergonomic measure	N
Individual (n = 32)	Improving awareness regarding ergonomics	21
	Worksite visits by an expert	2
	Physical activity programmes	5
	Training in working techniques, (*i.e.*, lifting technique)	3
	Personal protective equipment (*i.e.*, kneepads)	1

Physical (n = 27)	Ergonomic redesign and/or workstation modifications	18
	Manual handling aids (*i.e.*, lifting devices)	5
	Equipment and/or tools	4

Organisational (n = 7)	Installation of pause software	2
	Develop protocol to improve worker's health	1
	Restructuring management style	2
	Job rotation	2

To investigate whether the 66 prioritised ergonomic measures were actually implemented at the departments, the 81 implementers were sent a short questionnaire. A total of 65 of the implementers responded (80%). From the questionnaire, it appeared that the implementation status of three prioritised ergonomic measures was unknown (n = 1 individual, n = 2 physical). Therefore, this study evaluated the perceived implementation of 63 prioritised ergonomic measures (n = 31 individual; n = 25 physical; n = 7 organisational).

Implementers reported that altogether 34% of the prioritised ergonomic measures was implemented, 26% was partly implemented, and 40% was not implemented at the 18 departments. From the answers on the questionnaire, it was shown that within working groups implementers sometimes disagreed on the implementation status of the prioritised ergonomic measure. That is, one implementer perceived the measure as implemented, whereas another implementer within the same working group perceived the measure as not implemented. Table 4 presents the percentages of the perceived implementation stratified by type of ergonomic measure and department workload. In general, highest implementation rates were found for individual ergonomic measures (53%), and lowest implementation rates for organisational ergonomic measures (28%). At the light physical workload department, the implementation was 100%, but these results were obtained from only one department. Organisational ergonomic measures were most common at the departments with a mental workload and were in most cases 'partly' implemented (47%). Departments with a heavy physical workload most often prioritised physical ergonomic measures (n = 12), but the perceived implementation was low (16%). Departments with a mixed workload, and departments with a mental workload, most often prioritised individual ergonomic measures (n = 11). The perceived implementation between these two department types, however, varied largely (26% to 79%).

**Table 4:** Perceived implementation of the prioritised ergonomic measures according to the implementers (n = 65)

Ergonomic measures perceived as implemented	Type of ergonomic measure		
**All departments (n = 18)**	**Individual (n = 31)**	**Physical (n = 25)**	**Organisational (n = 7)**

Yes (%)	53	30	25

Partly (%)	21	26	47

No (%)	26	44	28

**Mental workload departments (n = 10)**	**Individual (n = 11)**	**Physical (n = 7)**	**Organisational (n = 5)**

Yes (%)	26	33	15

Partly (%)	32	41	46

No (%)	42	26	39

**Light physical workload departments (n = 1)**	**Individual (n = 1)**	**Physical (n = 2)**	**Organisational (N/A)**

Yes (%)	100	100	N/A

Partly (%)	0	0	N/A

No (%)	0	0	N/A

**Mixed workload departments (n = 3)**	**Individual (n = 11)**	**Physical (n = 4)**	**Organisational (N/A)**

Yes (%)	79	31	N/A

Partly (%)	17	13	N/A

No (%)	4	56	N/A

**Heavy physical workload departments (n = 4)**	**Individual (n = 8)**	**Physical (n = 12)**	**Organisational (n = 2)**

Yes (%)	44	16	50

Partly (%)	18	26	50

No (%)	38	58	0

N/A = not applicable

#### Participant level: dose received

According to the 833 workers who responded to the perceived implementation questions in the six-month follow-up questionnaire, 26% perceived the ergonomic measures as implemented, 36% as partly implemented, and 38% as not implemented at the departments. Table 5 presents the percentages of the perceived implementation of the ergonomic measures stratified by type of ergonomic measure and department workload. Among the 26% of the workers who perceived the ergonomic measures as implemented at the departments, the ergonomic measure was in 69% of the cases implemented at their workplace.

**Table 5:** Perceived implementation of the prioritised ergonomic measures according to the workers at the departments (n = 833)

Ergonomic measures perceived as implemented	Type of ergonomic measure		
**All departments (n = 18)**	**Individual (n = 31)**	**Physical (n = 25)**	**Organisational (n = 7)**

Yes (%)	28	26	19

No (%)	37	38	38

Don't know (%)	35	36	43

**Mental workload departments (n = 10)**	**Individual (n = 11)**	**Physical (n = 7)**	**Organisational (n = 5)**

Yes (%)	21	30	18

No (%)	44	42	52

Don't know (%)	35	28	30

**Light physical workload departments (n = 1)**	**Individual (n = 1)**	**Physical (n = 2)**	**Organisational (N/A)**

Yes (%)	40	32	N/A

No (%)	32	44	N/A

Don't know (%)	28	24	N/A

**Mixed workload departments (n = 3)**	**Individual (n = 11)**	**Physical (n = 4)**	**Organisational (N/A)**

Yes (%)	31	36	N/A

No (%)	36	37	N/A

Don't know (%)	33	27	N/A

**Heavy physical workload departments (n = 4)**	**Individual (n = 8)**	**Physical (n = 12)**	**Organisational (n = 2)**

Yes (%)	35	20	20

No (%)	29	36	67

Don't know (%)	36	44	13

N/A = not applicable

## Discussion

The Stay@Work study investigated whether PE is an effective method to prevent LBP and NP among workers. The aim of the current study was to evaluate the process of the Stay@Work PE programme implementation including the perceived implementation effectiveness of the prioritised ergonomic measures.

The results of this process evaluation showed that almost all department managers formed a working group and that a meeting was held with all working groups. Attendance rates of the working group meetings were good, and all working groups were successful in developing an implementation plan with prioritised risk factors for LBP and NP and prioritised ergonomic measures to prevent LBP and NP. Working group members were positive about the quality of the PE steps performed during the meeting, meeting duration, and the prioritised ergonomic measures. These opinions were not shared among the remaining workers at the departments. Attendance rates of the Stay@Work ergocoach training and the quality of the training were good. Workers at the departments were not satisfied with the implementation strategy used. Dissatisfaction may have occurred because workers at the departments were kept blind as to the study design and were thereby only marginally informed about the PE programme content and its aims. It is plausible that workers at the departments did not link the prioritised ergonomic measures to the PE programme and were therefore not sufficiently able to rate their satisfaction with the used method. Moreover, dissatisfaction among workers might have occurred because they were asked to report on the implementation of ergonomic measures that were not (always) applicable to their workplace. However, workers' satisfaction towards both the prioritised ergonomic measure and the method that was used to develop the ergonomic measures increased somewhat when the ergonomic measures were implemented at their workplace.

Overall, it can be concluded that the Stay@Work PE programme is a successful and feasible strategy to develop an implementation plan with prioritised risk factors for LBP and NP and prioritised ergonomic measures to prevent LBP and NP. It is more difficult, however, to draw conclusions regarding the implementation rates as there is no cut-off point to determine whether implementation was successful or has failed. Regarding the prevention of LBP and NP it can be suggested that every (extra) ergonomic measure implemented might be profitable [3,37,38], even when perceived implementation rates of 34% and 26% are derived. Future research should investigate whether the implementation rates found in this study are sufficient to reduce workload and thereby reduce LBP and NP prevalence among workers.

The perceived implementation rates found in our study differed from other studies on PE. For example Haukka *et al. *(2008) conducted a RCT on PE and MSD prevention and reported a perceived implementation rate of 80% (402 ergonomic changes) [22,39], although it remained unclear how they assessed whether an ergonomic measure was implemented. There are several explanations for the different implementation rates found in our study compared to other PE studies like the Haukka study.

In our study, individual ergonomic measures were prioritised most often, especially among departments with a mixed workload. The choice to prioritise and implement individual ergonomic measures seemed plausible, since the ergonomic measures were evaluated according to a set of common implementation criteria: low initial costs, not complex, compatible, visible, and feasible within three months. In line with other studies on PE, physical ergonomic measures were also prioritised frequently. However, other studies also found higher frequencies on organisational ergonomic measures [16,17,22,39,40]. The reason why fewer organisational ergonomic measures were prioritised in this study may be a result of the implementation criteria that were probably less applicable to evaluate organisational ergonomic measures. In addition, the implementation of physical or organisational measures is more complex, expensive, and time consuming to perform compared to individual ergonomic measures [30].

Another possible explanation involves the inconsistent answers on the implementation status of the prioritised ergonomic measure (yes/no/partly implemented). For example, within the same working group, two out of the five implementers reported that the prioritised ergonomic measure was implemented, whereas the remaining implementers reported that the ergonomic measure was not implemented. Such inconsistencies often made it impossible for the researchers to decide whether a measure really was implemented. More knowledge about the implementers' reasons for choosing a certain implementation status may have helped the researchers to make decisions about the implementation status of the prioritised ergonomic measures. However, due to the purpose of this study, no information on such reasons was collected. Furthermore, inconsistency may have been caused by the high number of 'yes, partly implemented' answers. In our questionnaire that was sent to the implementers, we did not specifically define the term 'yes, partly implemented'. However, from the information obtained from the questionnaire we suspect that some implementers chose 'yes, partly implemented' when they discovered that it was more beneficial to implement a prioritised ergonomic measure for only a subgroup of workers rather than for all workers at the intervention department. Other implementers appear to have chosen 'yes, partly implemented' when the implementation of the prioritised ergonomic measure was in progress but had not been completely realised yet. For example, in case of the implementation of a lifting device, implementers ordered the device; however, the lifting device was not yet being used at the workplace.

Finally, although several explanations for the modest implementation have been discussed, it is possible that other unmeasured factors might have occurred during the implementation period (*e.g.*, hierarchy, poor management support, lack of assistance, or financial problems) thereby hampering implementation [41]. For example, it is plausible that a lack of financial resources may have hampered the implementation of ergonomic measures. This is because most working groups were conducted in 2008 -- a time when many Dutch companies experienced the consequences of the international financial downturn. Moreover, different implementation factors may be present or absent at different stakeholder levels (*i.e.*, individual professional, worker, societal, or organisational level) [14]. More in-depth knowledge on implementation factors and their stakeholder level can help researchers to improve ergonomic interventions. Therefore, to further improve the implementation of this or future PE programme(s), it may be helpful to explore what factors hampered or facilitated the implementation of ergonomic measures.

### Strengths and weaknesses of the process evaluation

No other study implemented PE on such a large scale and among departments with different type of workloads. Furthermore, this process evaluation study collected extensive data on the perceived implementation. In doing so, this study attempted to estimate the efficiency of the PE programme and the implementation strategies. The existing literature suggests that the use of informational material alone is not sufficient to induce a behavioural change (*i.e.*, use of ergonomic measures). More active strategies such as toolkits and local opinion leaders should be used to disseminate information [13]. Therefore, a strength of this study was that not only informational materials but also ergocoaches (opinion leaders) trained to inform, motivate, and instruct their co-workers on the ergonomic measures. Further, data were collected from different stakeholders at different levels which provided a better understanding of how the different stakeholders experienced the PE programme and the implementation strategies.

A weakness of this study is that selection bias may have occurred because not all implementers and not all workers at the department responded to their questionnaires. Furthermore, the accuracy of the method that was used to measure implementation is debatable. All workers at the department were asked whether the prioritised ergonomic measures were implemented. Due to the variety of task groups within departments, it may be that some workers were asked to report on implementations that were not meant for their workplace. The same goes for the implementers, who during the implementation of the ergonomic measures may have discovered that a prioritised ergonomic measure was more beneficial for a subgroup of workers rather than for the whole department. This may have led to misinterpretations of the concept of implementation and may have resulted in inconsistent answers on the questionnaires. A possible solution to overcome such inconsistencies and to increase the validity of the answers provided by the implementers is to arrange control visits by an ergonomist [42]. Finally, the role of the ergonomist in the current study was restricted to guiding the working group meeting. In line with the PE literature [43], working group members themselves were responsible for the implementation of the prioritised ergonomic measures. Although working group members were allowed to seek help from other professionals during the implementation period, no information on which professionals were consulted was collected. It is, however, plausible that more assistance and cooperation from the ergonomist, other professionals (*i.e.*, suppliers, technicians, and purchase) and the management to realise implementation, might indeed have led to higher implementation rates.

## Summary

The results of this process evaluation showed that PE can be a feasible and successful strategy to develop an implementation plan with prioritised risk factors for LBP and NP and prioritised ergonomic measures to prevent LBP and NP. Moreover, recruitment, reach, fidelity, and satisfaction towards the PE programme were good. The same was found for the Stay@Work ergocoach training. Despite the positive rating of the PE programme and the ergocoach training, the implementation of the prioritised ergonomic measures was lower than expected. Further research is needed to develop and test ways to more optimally implement PE programmes in order to reduce work-related injuries and to promote worker well-being.

## Competing interests

The authors declare that they have no competing interests.

## Authors' contributions

All authors contributed to the design of the study. MTD is the principle researcher and was responsible for the data collection and data analyses. JRA contributed to the conception and the design of the study and coordinated the study. KIP, JRA, PMB, and AJvdB supervised the study. All authors contributed to writing up of this paper and approved the final manuscript.
